# Editorial: Cellular CNS repair strategies, technologies and therapeutic developments

**DOI:** 10.3389/fncel.2023.1200639

**Published:** 2023-04-28

**Authors:** Tim-Henrik Bruun, Jorg Dietrich, Anna Klingseisen, Ulrich Bogdahn

**Affiliations:** ^1^Velvio GmbH Regensburg, Regensburg, Germany; ^2^Harvard Medical School Boston, Boston, MA, United States; ^3^UK Dementia Research Institute, University of Edinburgh, Edinburgh, United Kingdom; ^4^Department of Neurology, University Hospital Regensburg, Regensburg, Germany

**Keywords:** neurodegeneration, drug-development, CNS-repair, biomarker, study-design in CNS

The central nervous system (CNS) plays a key role in controlling and coordinating the body's functions and movements. Damage to the CNS may have devastating effects, including paralysis, sensory loss, and cognitive impairment. However, recent advances in cellular CNS repair strategies have brought new hope for patients suffering from CNS injuries or neurodegenerative disorders. The Research Topic “*Cellular CNS repair strategies, technologies and therapeutic developments*” featured in Frontiers in Neuroscience, highlights several novel aspects and advances in this exciting field.

The special Research Topic includes contributions from 20 committed scientists and researchers from around the world, providing valuable insights into some of the latest technologies and therapeutic developments in cellular CNS repair. The articles range from basic science to clinical applications, covering a broad spectrum of topics that are crucial for advancing our understanding of CNS repair.

One of the main themes that emerge from the Research Topic is the use of stem cells for CNS repair. Stem cells have the potential to differentiate into various cell types, making them ideal for repairing damaged or diseased tissue. In their article, Feng et al. discuss the use of neural stem cells in the treatment of spinal cord injury (SCI), demonstrating the promising results of preclinical studies.

Another major theme in the Research Topic is the role of neurotrophic factors for CNS repair. Neurotrophic factors are proteins that promote the growth and survival of neurons and some non-neuronal cells, and they have been shown to play a crucial role in the regeneration of damaged CNS tissue (Chen et al.). In their article, Olmstedt et al. describe how the outcome of neural cell-based therapies in SCI might be improved.

Several articles in this Research Topic also explore the use of novel technologies for CNS repair. For example, Feng et al. discuss the use of immune microenvironment and tissue engineering strategies for CNS repair, highlighting the potential of this approach for improving the efficacy and specificity of SCI therapies. Jurek et al. used a 3D culture model and found that Oxytocin (OXT) decreases cellular migration but increases cell-cell contacts and therefore improves nutrient supply, which might have implications for degenerating CNS disorders and tumor formation in various tissues.

The Research Topic also includes articles that focus on clinical applications of cellular CNS repair strategies. For example, Wu et al. summarizes cell types involved in the repair process and the common repair mechanisms of spinal cord injury, emphasizing the importance of clinical trial design and patient selection for the success of SCI therapies. Shi et al. also describe the use of miRNA targets for traumatic brain injury (TBI), demonstrating a potential target for the treatment of TBI related dysfunction.

Overall, the Research Topic “*Cellular CNS repair strategies, technologies and therapeutic developments*” provides valuable examples of the current state of research in this exciting field. The featured contributions highlight several examples of the significant progress that has been made in recent years, as well as the challenges that lie ahead. By bringing together researchers from diverse disciplines and perspectives, the Research Topic provides a potential roadmap for future research in this critical area.

It is worth noting that the Research Topic also sheds light on some of the ethical considerations surrounding the use of cellular CNS repair strategies. As the field continues to advance, it is important to ensure that these therapies are developed and deployed in an ethical and responsible manner. Mueller et al. discusses the importance of considering the potential risks and benefits of stem cell therapies, as well as the need for standardized protocols in the manufacturing and testing of stem cell products.

## Conclusion

In conclusion, the Research Topic “Cellular CNS repair strategies, technologies and therapeutic developments” is a valuable resource for researchers, clinicians, and policymakers interested in the field of CNS repair. This special edition and collection of articles provides a comprehensive overview of the latest technologies and therapeutic developments, as well as highlighting existing challenges and future perspectives. As the field continues to advance, there is increasing evidence that cellular CNS repair strategies will play an increasingly important role in the treatment of CNS injuries and degenerative disorders, bringing new hope to patients around the world, and bringing to our attention that we never had been dreaming of repairing the brain and spinal cord.

## Author contributions

T-HB wrote the first draft of the manuscript. All authors contributed to manuscript revision, read, and approved the submitted version.

